# Anesthetic outcomes in robotic kidney transplantation: a retrospective cohort analysis

**DOI:** 10.1007/s11701-026-04168-w

**Published:** 2026-11-13

**Authors:** Jessalyn Guo-Yee Yam, Lauren Sydney Huang, Christopher Chiang, Alexandra Barabanova, Ivo G. Tzvetanov, Pierpaolo Di Cocco, Mario Spaggiari, Stephen Bartlett, Jorge A. Almario-Alvarez, Enrico Benedetti, Michael R. Fettiplace

**Affiliations:** 1 Department of Anesthesiology, University of Illinois Chicago College of Medicine, Chicago, IL USA; 2 Department of Medical Education, Georgetown University, Washington, DC USA; 3 Department of Surgery, University of Illinois Chicago College of Medicine, Chicago, IL USA; 4 Present Address: Department of Surgery, Loyola University Chicago, Chicago, USA

**Keywords:** ERAS (enhanced recovery after surgery), Perioperative pain management, Postoperative opioid consumption, Robotic kidney transplantation

## Abstract

Kidney transplantation is the definitive treatment for end-stage renal disease. Within the last decade, robotic-assisted kidney transplants have emerged as a promising alternative to the traditional open approach, but the impact on postoperative pain is unclear. We conducted a propensity-matched retrospective study to investigate postoperative pain control between open and robotic kidney transplant in adults at the University of Illinois Hospital from September 2020 to September 2025. The primary outcome was opioid consumption within 30 days after surgery. Secondary outcomes included the highest pain score within 72 hours of surgery, intraoperative opioid consumption, length of stay, and total hospital length of stay. Of 1379 patients, significant differences in baseline characteristics were observed between the open (*n* = 1234) and robotic (*n* = 145) approaches for age, weight and BMI. Propensity matching resulted in 127 pairs with no significant difference in opioid consumption within 30 days of surgery, intraoperative milligram morphine equivalents, highest pain score in recovery within 72 hours, and length of stay. Robotic cases were longer with less blood loss and required a smaller volume of packed red blood cell transfusions. Multivariate linear regression of 30-day morphine equivalents demonstrated a reduction in patients who were immediately extubated following completion of surgery. Robotic transplants resulted in similar postoperative opioid consumption and pain scores to open transplants but were longer and less bloody. Prolonged intubation after surgery contributed to opioid use. Our results contradict prior literature, which has reported reduced pain and opioid use with robotic approaches.

**Supplementary Information:**

The online version contains supplementary material available at https://doi.org/10.1007/s11701-026-04168-w.

## Introduction

Kidney transplantation is the definitive treatment for end‑stage renal disease (ESRD), providing superior survival and quality of life compared to long‑term dialysis [1]. Traditionally, transplants are performed via an open surgical approach, which requires extensive tissue dissection [2]. The approach contributes to postoperative pain and opioid analgesic requirements, presenting a critical challenge for perioperative care and pain management [3].

Over the past decade, robotic‑assisted kidney transplantation has revolutionized transplantation, driven by technical advantages, including smaller incisions, enhanced visualization, and improved ergonomics for the surgeon [4]. Kidney transplant programs in the United States frequently use Body Mass Index (BMI) cutoff of 40 kg/m^2^ for transplant eligibility since obesity in open transplantation contributes to increased cardiometabolic comorbid conditions, such as cardiovascular complications and diabetes, increased risk of graft loss, and increased risk of mortality [5]. The introduction of robotic transplants offers an option for obese patients who previously did not qualify for open surgery or were considered too overweight to undergo transplantation at other hospitals [2, 3, 6]. Additionally, the less invasive nature can reduce tissue trauma, minimize intraoperative blood loss, and accelerate postoperative recovery, which benefits patients with higher surgical risk or more comorbidities [7]. Since the first robotic intra-abdominal kidney transplant in 2010, the technique has evolved, with broader adoption across specialized centers [2, 6, 8]. These advancements have expanded access to minimally invasive transplantation for complex and high-risk populations, including those with morbid obesity and higher metabolic comorbidity burden who were historically excluded from open procedures [9].

Prior reports indicate superior outcomes with robotic compared to open transplantation, including reduced postoperative complications, readmission rates, and wound morbidity [10, 11]. Despite these advances, the perioperative anesthetic outcomes in robotic transplantation remain incompletely characterized. This is an important question since perioperative opioid exposure is associated with increased risk of mortality, graft loss, post-transplant complications, opioid dependence, and hospital readmission following transplantation [12]. Further, comparative data between robotic and open transplant remain limited [13, 14]. As such, we chose to evaluate outcomes between open and robotic transplants in a single-center retrospective, propensity-matched study. We hypothesized that robotic transplant would lead to lower postoperative opioid requirements and reduced pain scores due to its minimally invasive nature.

## Methods

### Study design and population

**Fig. 1:**
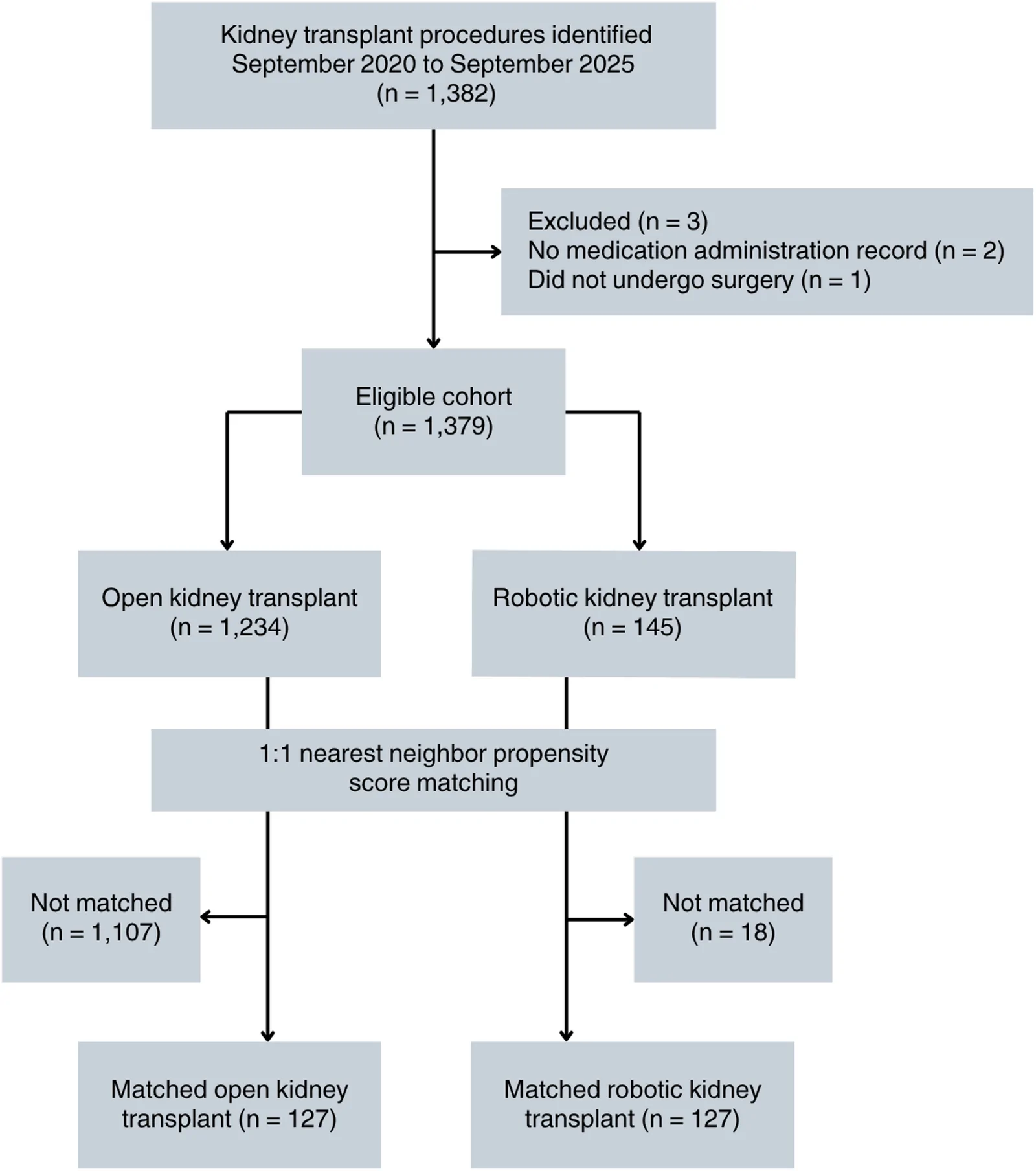
Participant flow from procedure identification through propensity score matching

Institutional Review Board (IRB Study 2025 − 0991) approval and waiver of Health Insurance Portability and Accountability Act (HIPAA) authorization was granted for this study. We conducted a propensity-matched retrospective cohort study of adults (≥ 18 years old) who underwent kidney transplantation at the University of Illinois Hospital from September 2020 to September 2025 (Fig. 1). Exclusion criteria were patients younger than 18 years of age, pregnant women, cognitively impaired adults, and/or prisoners. We extracted data using a combination of manual chart review and SlicerDicer on EPIC (EPIC Systems, Verona, Wisconsin), our institution’s electronic health record system. Demographics, including age, sex, race, weight, BMI, American Society of Anesthesiologists (ASA) physical status, and pre-existing comorbidities, such as hypertension, hyperlipidemia, diabetes mellitus, coronary artery disease, congestive heart failure, obstructive sleep apnea, and anemia, were collected.

Perioperative variables included procedure duration (in minutes), estimated blood loss (EBL, in mL), number of packed red blood cell (pRBC) transfusion units administered, total intraoperative opioid administration expressed in morphine milligram equivalents (MME), and extubation status in the operating room were collected. Postoperative variables included cumulative postoperative MME at 6, 24, and 48 h after surgery, highest documented pain score in recovery (using either visual analog scale or FACES pain scale) within the first 72 h postoperatively, ICU length of stay (in days), recovery length of stay (in days), time to first readmission (in days), total inpatient MME during the 30-day postoperative period, outpatient opioid prescriptions within the 90-day postoperative period, delayed graft function represented by dialysis within 7 days following transplant [15], and dialysis dependence within one year as markers of early graft failure [16]. All data were abstracted by trained reviewers using predefined definitions and entered into University of Illinois Chicago’s Box (Box Inc., Redwood City, CA), a secure research database. Opioid doses for total inpatient MME during the 30-day postoperative period were converted to MME using standardized conversion factors (**Supplementary Table 1**) [17, 18]. The primary outcome was 30-day post-operative opioid use, which captures all systemic opioids administered during inpatient care within the first 30 postoperative days. Secondary outcomes included highest pain score, intraoperative opioid use, procedural characteristics, and various clinical outcomes, including length of stay, 90-day outpatient opioid prescriptions, and graft function.

### Statistical analysis

All statistical analyses were conducted using GraphPad Prism Version 10.5.0 (673) and R version 2026.01.0 + 392 (R Foundation for Statistical Computing, Vienna, Austria). Data were analyzed with a significance threshold of *p* < 0.05. Before matching, age was compared using the independent-samples t-test, weight and body mass index using the Mann-Whitney U test, and binary and ordinal variables using Fisher’s exact test. A propensity score for receiving robotic surgery was estimated for each patient using a logistic regression model. This model included demographic, anthropometric, and clinical variables that were selected based on clinical relevance: age, sex, race, weight, BMI, ASA physical status, hypertension (HTN), hyperlipidemia (HLD), diabetes mellitus (DM), coronary artery disease (CAD), congestive heart failure (CHF), obstructive sleep apnea (OSA), and anemia (Table 1, **Supplementary Table 2**). Nearest-neighbor matching without replacement was conducted in a 1:1 fashion using a caliper width of 0.2 to prevent poor matches. Patients whose propensity scores fell outside the caliper were excluded from the matched cohort. Covariate balance before and after matching was assessed using standardized mean differences (SMDs), with SMD < 0.1 considered acceptable balance.

**Table 1:** Demographics and clinical characteristics before and after propensity-score matching

Preoperative characteristics	Before matching				After matching			
	Total (*N* = 1379)	**Open** (*n* = 1234)	**Robotic** (*n* = 145)	SMD	**Total** (*N* = 254)	**Open** (*n* = 127)	**Robotic** (*n* = 127)	SMD
Age (years), mean (SD)	50.7 (12.5)	51.3 (12.7)	45.6 (9.9)	−0.57	46.4 (9.8)	46.3 (10.2)	46.4 (9.5)	0.02
Sex, Male, % (*n*)	60.6 (835)	60.8 (750)	58.6 (85)	−0.04	57.9 (147)	59.8 (76)	55.9 (71)	−0.08
Race, % (*n*)								
Black or African American	41.0 (565)	39.2 (484)	55.9 (81)	0.34	55.9 (142)	55.9 (71)	55.9 (71)	0.00
White	49.9 (688)	51.1 (630)	40.0 (58)	−0.23	40.2 (102)	40.2 (51)	40.2 (51)	0.00
Other	9.1 (126)	9.7 (120)	4.1 (6)	−0.28	3.9 (10)	3.9 (5)	3.9 (5)	0.00
Weight (kg), median (IQR)	85.0 (69.6-106.3)	81.6 (68.0-101.2)	121.6) (106.3-136.2)	1.68	117.9 (104.5-132.9)	117.3 (104.7-133.2)	119.1 (104.9-132.7)	0.00
BMI (kg/m²), median (IQR)	30.3 (25.2–36.2)	28.9 (24.8–34.4)	40.8 (36.7–44.4)	1.87	40.1 (36.4–43.4)	40.1 (36.2–43.6)	40.0 (36.5–43.1)	0.05
ASA Score, % (*n*)								
II	0.5 (7)	0.4 (5)	1.4 (2)	0.08	0.4 (1)	0.0 (0)	0.8 (1)	0.07
III	87.3 (1204)	87.2 (1076)	88.3 (128)	0.03	89.0 (226)	89.0 (113)	89.0 (113)	0.00
IV	12.2 (168)	12.4 (153)	10.3 (15)	−0.07	10.6 (27)	11.0 (14)	10.2 (13)	−0.03
Comorbidities, % (*n*)								
HTN	28.7 (396)	29.7 (366)	20.7 (30)	−0.22	20.1 (51)	19.7 (25)	20.5 (26)	0.02
HLD	33.4 (461)	33.0 (407)	37.2 (54)	0.09	37.4 (95)	37.8 (48)	37.0 (47)	−0.02
DM	49.5 (682)	49.1 (606)	52.4 (76)	0.07	54.7 (139)	55.1 (70)	54.3 (69)	−0.02
CAD	19.4 (268)	20.3 (250)	12.4 (18)	−0.23	9.8 (25)	6.3 (8)	13.4 (17)	0.22*
CHF	16.3 (225)	16.7 (206)	13.1 (19)	−0.11	16.1 (41)	17.3 (22)	15.0 (19)	−0.07
OSA	19.2 (265)	16.5 (204)	42.1 (61)	0.52	37.8 (96)	37.8 (48)	37.8 (48)	0.00
Anemia	44.5 (613)	44.2 (546)	46.2 (67)	0.04	45.7 (116)	45.7 (58)	45.7 (58)	0.00

ASA = American Society of Anesthesiologists; BMI = body mass index; CAD = coronary artery disease; CHF = congestive heart failure; DM = diabetes mellitus; HLD = hyperlipidemia; HTN = hypertension; OSA = obstructive sleep apnea; SD = standard deviation; SMD = standardized mean difference. Data are mean (SD), median (IQR), or % (n). SMD calculated for continuous and binary covariates using the standard deviation of the pre-matching robotic group as a common denominator; |SMD| < 0.10 indicates adequate balance

Before matching: independent-samples t-test (age), Wilcoxon rank-sum test (weight, BMI), Fisher’s exact test (categorical). After matching: paired t-test (age), Wilcoxon signed-rank test (weight, BMI), McNemar’s test (binary covariates; exact binomial form where < 25 discordant pairs), Stuart–Maxwell test of marginal homogeneity (race, ASA)

SMDs for all covariates are reported in Supplementary Table 2

*Residual imbalance after matching (|SMD| ≥ 0.10). Sensitivity analyses adjusting for coronary artery disease are reported in Supplementary Table 3

Following matching, paired t-test for age, the Wilcoxon signed-rank test for weight and body mass index, McNemar’s test for binary variables (exact binomial form where fewer than 25 discordant pairs were observed), and the Stuart-Maxwell test of marginal homogeneity for race and ASA physical status. The matched dataset was then used for all downstream analyses to estimate treatment effects while minimizing measured confounding. A complete-case analysis was conducted for all variables included in Table 2.

**Table 2:** Intraoperative variables & clinical outcomes after matching

Variables	Total (*n* = 254)	Open (*n* = 127)	Robotic (*n* = 127)	*p*-value
**Intraoperative variables**				
Procedure Length (min)ᵃ, *n* = 121 pairs	248.5 (211.0-299.2)	223.0 (194.5–277.0)	274.0 (235.0-316.0)	< 0.0001
Estimated Blood Loss (mL)^b^, *n* = 126 pairs	100.0 (50.0-200.0)	100.0 (50.0-225.0)	90.0 (50.0-117.5)	0.001
PRBC Transfusion Volume (mL), *n* = 127 pairs	0.0 (0.0-300.0)	0.0 (0.0-450.0)	0.0 (0.0-300.0)	0.04
Intraoperative Opioid Consumption (MME)^c^, *n* = 119 pairs	46.0 (38.0-61.8)	46.0 (38.0–60.0)	46.0 (38.0–68.0)	0.14
**Clinical outcomes**				
Opioid Consumption at 6 h (MME) ^c^, *n* = 119 pairs	63.3 (30.2–93.5)	57.1 (17.2–86.8)	71.4 (38.0-108.4)	0.17
Opioid Consumption at 24 h (MME)^c^, *n* = 119 pairs	225.4 (64.0-370.6)	181.9 (46.1–353.0)	245.4 (114.3-408.8)	0.14
Opioid Consumption at 48 h (MME)^c^, *n* = 119 pairs	252.5 (75.9–461.0)	195.7 (57.0-464.2)	270.4 (119.0-458.6)	0.73
Total Inpatient Opioid Consumption within 30 Days (MME)^d^, *n* = 126 pairs	434.0 (277.5–599.0)	383.0 (254.0–595.2)	454.0 (306.0–599.0)	0.47
Highest Pain Score within 72 Hours^e^, *n* = 85 pairs	5.0 (3.0–7.0)	5.0 (3.0-7.2)	5.0 (3.0–7.0)	0.69
Remained Intubated After Procedure, % (n), *n* = 127 pairs	82.7 (210)	78.0 (99)	87.4 (111)	0.03
ICU Length of Stay (days)^f^, *n* = 116 pairs	4.3 (3.4–5.8)	4.4 (3.7–5.6)	4.2 (3.2–5.8)	0.99
Hospital Length of Stay (days), *n* = 127 pairs	7.0 (6.0–9.0)	7.0 (6.0–9.0)	7.0 (5.5-9.0)	0.36
Time to Readmission (days)^g^, *n* = 44 pairs	9.0 (4.0–19.0)	9.0 (4.0–19.0)	9.0 (4.0–21.0)	0.91
Outpatient Opioid Use within 90 Days^h^, % (n)	11.8 (30)	14.1 (18)	9.4 (12)	
Delayed Graft Function (HD within 7 days)^h^, % (n)	9.4 (24)	9.4 (12)	9.4 (12)	
Graft Failure (HD beyond 365 days)^h^, % (n)	1.6 (4)	0.3 (1)	1.1(3)	

Data are median (interquartile range) or % (n). Continuous variables were compared using the paired t-test; binary variables were compared using McNemar’s test. Pair counts reflect matched pairs with non-missing data for both members

ICU = intensive care unit; MME = morphine milligram equivalents; PRBC = packed red blood cells; HD = hemodialysis

^a^Six of 127 matched pairs excluded due to missing data

^b^ One of 127 matched pairs excluded due to missing data

^c^Intraoperative and 6, 24, and 48 h opioid consumption: Eight of 127 matched pairs excluded due to missing data. Obtained as pre-calculated morphine milligram equivalents from the institutional data extract; underlying conversion method was not available to the investigators

^d^One of 127 matched pairs excluded due to missing data. Calculated from medication administration records using NIH HEAL 2024 conversion factors for oral formulations and Nielsen et al. factors for parenteral formulations; expressed in oral morphine milligram equivalents. See Supplementary Table 1 for conversions

^e^Forty-two of 127 matched pairs excluded due to missing data

^f^Eleven of 127 matched pairs excluded due to missing data

^g^Eighty-three of 127 matched pairs excluded

^h^An insufficient number of complete matched pairs was available for statistical analysis; these outcomes are reported as % (n) only

A sensitivity analysis of the primary matched comparison, total inpatient opioid consumption within 30 days was log-transformed and modeled using multivariable linear regression (**Supplementary Table 3**). Baseline covariates included surgical approach and pre-surgery characteristics (age, sex, race, weight, BMI, and ASA physical status). To assess whether residual imbalance in CAD influenced our findings, this model was refitted with CAD added, and subsequently with all remaining comorbidities (HTN, HLD, DM, CHF, OSA, and anemia) added. Because matching induces dependence between the members of each pair, the base model was also refitted with standard errors clustered on matched pair, as recommended for matched designs [19]. Finally, to capture the influence of adjusting for variables measured intraoperatively and postoperatively, the model was refitted with procedure duration, intraoperative MME, estimated blood loss, extubation status, and highest postoperative pain score within 72 h, consistent with the model used in Table 3. Estimates for surgical approach are reported as ratios of geometric means with 95% confidence intervals, together with the number of patients and complete matched pairs contributing to each model.

**Table 3:** Multivariate linear regression of total inpatient opioid consumption within 30 days (MME)

Variable	Ratio of geometric means (95% CI)	% change in 30d MME (95% CI)	*p*-value
Intercept	90.18 (29.51 to 275.54)	8917.9% (2851.4% to 27453.8%)	< 0.0001
Age (years)	1.00 (0.99 to 1.01)	0.0% (− 1.1% to 1.2%)	0.94
Sex			
Male	1.17 (0.87 to 1.57)	16.7% (− 13.3% to 57.2%)	0.31
Female	1 [Reference]	1 [Reference]	NA
Race			
Black	1 [Reference]	1 [Reference]	NA
Other	1.39 (0.74 to 2.59)	38.7% (− 25.8% to 159.2%)	0.30
White	0.98 (0.77 to 1.25)	−1.8% (− 22.7% to 24.8%)	0.88
Weight (kg)	1.00 (0.99 to 1.01)	0.2% (− 0.8% to 1.1%)	0.75
BMI (kg/m²)	1.03 (0.99 to 1.06)	2.8% (− 0.7% to 6.4%)	0.12
Procedure Type			
Robotic	0.99 (0.78 to 1.27)	−0.5% (− 22.1% to 27.1%)	0.97
Open	1 [Reference]	1 [Reference]	NA
ASA Physical Status			
ASA Score, II	0.84 (0.18 to 3.84)	−16.3% (− 81.7% to 283.8%)	0.82
ASA Score, III	1 [Reference]	1 [Reference]	NA
ASA Score, IV	1.17 (0.83 to 1.66)	17.5% (− 16.8% to 65.9%)	0.36
Procedure Length (min)	1.00 (1.00 to 1.00)	0.2% (0.0% to 0.3%)	0.04
Intraoperative MME (mg)	1.00 (1.00 to 1.00)	0.0% (− 0.4% to 0.4%)	0.96
EBL (per 100 mL)	0.95 (0.89 to 1.02)	−5.0% (− 11.4% to 2.0%)	0.15
Extubated in OR?			
Yes	0.47 (0.35 to 0.64)	−52.8% (− 65.3% to − 35.8%)	< 0.0001
No	1 [Reference]	1 [Reference]	NA
Highest Pain Score within 72 h	0.98 (0.95 to 1.02)	−1.6% (− 5.2% to 2.0%)	0.38

This analysis was exploratory and was not intended to estimate the effect of surgical approach. Several covariates occur during or after surgery and may lie on the causal pathway between surgical approach and opioid consumption; the coefficient for procedure type should not be interpreted as a treatment effect

The model was fitted on 195 patients, comprising 76 complete matched pairs and 43 patients whose matched counterpart was excluded for missing covariate data

Total inpatient opioid consumption within 30 days was log-transformed prior to multivariable linear regression to improve normality of residuals. Estimates are presented as exponentiated coefficients, representing ratios of geometric means and percent difference. A ratio above 1.00 indicates higher opioid consumption

ASA = American Society of Anesthesiologists; BMI = body mass index; EBL = estimated blood loss; MME = morphine milligram equivalents

An exploratory analysis of postoperative opioid consumption during the first 30 days of hospitalization was evaluated using multiple linear regression. To improve residual normality and satisfy model assumptions, total inpatient opioid consumption within 30 days was log-transformed prior to analysis. Covariates included age, sex, race, weight, BMI, procedure type, ASA physical status, procedure duration, intraoperative MME, extubation status at the conclusion of the procedure, EBL, and highest postoperative pain score within 72 h. Model assumptions were assessed using diagnostic testing and visual inspection of residual plots, including quantile–quantile plots for normality and residual-versus-fitted plots for homoscedasticity. Regression coefficients were back-transformed and reported as ratio of geometric means and percent change in postoperative 30-day MME. EBL was scaled such that estimates represent the percent change in postoperative 30-day MME per 100 mL increase in blood loss (Table 3). Values were not truncated, winsorized, or otherwise adjusted and represent accurate administrations of opioid utilization.

Generative AI, including Grammarly, was used to improve the fluency and clarity in the introduction and discussion sections. Claude (Anthropic) was used to assist with drafting and debugging of R code used for the statistical analyses. All code was reviewed and verified by authors. No data was entered into these tools, consistent with recommendations in the regional anesthesia and pain medicine community [20]. ​ 

## Results

### Patient characteristics

Of 1379 patients included in this study, we observed significant differences in baseline characteristics between the open (*n* = 1234) and robotic transplant (*n* = 145) groups (Table 1, **Supplementary Table 2**) for age (mean ± SD, 51.3 ± 12.7 vs. 45.6 ± 9.9 years, SMD − 0.57, *p* < 0.001), weight (median [IQR], 81.6 [68.0–101.2] vs. 121.6 [106.3–136.2] kg, SMD 1.68, *p* < 0.001) and BMI (28.9 [24.8–34.4] vs. 40.8 [36.7–44.4] kg/m^2^, SMD 1.87, *p* < 0.001). This indicated a younger, heavier, and more obese population in the robotic approach. Obstructive sleep apnea was more prevalent among robotic recipients (16.5% [204] vs. 42.1% [61], SMD 0.52, *p* < 0.001), and racial distribution differed between groups (*p* < 0.001), with a greater proportion of Black or African American patients undergoing robotic transplantation (39.2% [484] vs. 55.9% [81], SMD 0.34). Hypertension (29.7% [366] vs. 20.7% [30], SMD − 0.22, *p* = 0.03) and coronary artery disease (20.3% [250] vs. 12.4% [18], SMD − 0.24, *p* = 0.03) were less prevalent in the robotic group.

Propensity matching resulted in 127 pairs (254 total) with similar baseline characteristics and comorbidities (Table 1, **Supplementary Table 2**), with 15 of 16 covariates achieving adequate balance. Patients were evenly distributed among sex and race, though they were predominantly ASA physical status III (% [n], 89.0 [113] vs. 89.0 [113], SMD 0.00, *p* = 0.59) and obese (BMI, 40.1 [36.2–43.6] vs. 40.0 [36.5–43.1] kg/m^2^, SMD 0.05, *p* = 0.61). Approximately half of the patient population had diabetes mellitus (55.1% [70] vs. 54.3% [69], SMD − 0.02, *p* = 0.90) and anemia (45.7% [58] vs. 45.7% [58], SMD 0.00, *p* = > 0.99). CAD remained imbalanced after matching, with a higher prevalence among robotic recipients (6.3% [8] vs. 13.4% [17], SMD 0.22, *p* = 0.09). Sensitivity analyses adjusting for CAD demonstrated that the residual imbalance does not account for our findings (**Supplementary Table 3**).

### Outcomes following propensity matching

Within the matched cohort, there were no significant differences in total inpatient MME within 30 days postoperatively (383.0 [254.0-595.2] vs. 454.0 [306.0-599.0] MME, *p* = 0.47) or highest recorded postoperative pain score between groups (5.0 [3.0-7.2] vs. 5.0 [3.0–7.0], *p* = 0.69). Similarly, postoperative opioid utilization at 6, 24, or 48 h was identical. We were unable to elicit meaningful conclusions about outpatient opioid use within 90 days following the procedure due to insufficient matched pairs (Table 2).

Robotic transplant was associated with a longer procedure length (223.0 [194.5–277.0] vs. 274.0 [235.0-316.0] minutes, *p* < 0.0001), lower estimated blood loss (EBL, 100.0 [50.0-225.0] vs. 90.0 [50.0-117.5] mL, *p* = 0.001), and lower pRBC transfusion volume (0.0 [0.0-450.0] vs. 0.0 [0.0-300.0], *p* = 0.04). However, there was no difference in intraoperative MME (46.0 [38.0–60.0] vs. 46.0 [38.0–68.0] MME, *p* = 0.14).

Robotic patients were more likely to remain intubated at the conclusion of the procedure (78.0% [99] vs. 87.4% [111], *p* = 0.03). There were no significant differences in patients’ ICU length of stay, overall hospital length of stay, or time to readmission. Outpatient opioid prescriptions within 90 days of surgery were recorded in 14.2% [18] of open and 9.4% [12] of robotic recipients. Delayed graft function, defined as hemodialysis within 7 days of transplantation, occurred in 9.4% [12] of patients in each group. Graft failure, defined as hemodialysis beyond 365 days, occurred in 0.8% [1] of open and 2.4% [3] of robotic recipients. We were unable to draw meaningful conclusions on these variables due to an insufficient number of pairs following propensity matching. These values are reported descriptively and were not compared statistically.

### Multiple linear regression of 30-day MME

In an exploratory multivariable linear regression including 195 patients (76 complete matched pairs), two variables were associated with total inpatient opioid consumption within 30 days (Table 3). Extubation at the conclusion of the procedure was independently associated with a 52.8% lower 30-day MME (ratio of geometric means 0.47, 95% CI: 0.35 to 0.64, *p* < 0.0001. Longer procedure length was associated with modestly higher consumption, with a 0.2% increase in postoperative 30-day MME for each additional minute (95% CI: 0.0% to 0.3%, *p* = 0.04). Procedure type was not associated with 30-day opioid consumption in this model (ratio of geometric means 0.99, 95% CI: 0.78 to 1.27, *p* = 0.97). No other covariate was associated with the outcome, including age, sex, race, weight, BMI, ASA physical status, intraoperative MME, estimated blood loss, and highest postoperative pain score within 72 h.

## Discussion

In this propensity-matched retrospective cohort study, robotic transplant exhibited similar recovery characteristics to the open cohort, including similar postoperative opioid consumption, similar pain scores, and a similar hospital length of stay. These results demonstrate that robotic cases recover at rates similar to open approaches but still experience postoperative pain, despite the less invasive technique. Multivariable analysis confirmed that the surgical approach was not an independent predictor of postoperative opioid use. Instead, postoperative intubation emerged as the most significant correlate of postoperative opioid use. Additionally, procedure length correlated with opioid use. Both correlates highlight opportunities for future interventions to accelerate patient recovery and pain control with Enhanced Recovery After Surgery (ERAS) protocols.

An increasing number of studies in recent years have demonstrated that robotic transplantation is a viable alternative to open transplant, with several reports suggesting reduced postoperative pain, opioid requirements, and perioperative morbidity [10, 21, 22]. Prior work frequently attributes the benefits to the minimally invasive nature of robotic transplant, including smaller incisions and reduced tissue trauma [23]. Many studies are limited by selective inclusion criteria, often focusing on lower-risk patients or excluding those with higher BMI and complex comorbidities, which limits the generalizability of their findings [21, 24]. A single randomized controlled trial compared robotic and open kidney transplantation and found that robotic surgery resulted in higher postoperative opioids due to the more predictable pain in open surgery [25]. Our propensity-matched design with a mean BMI of 40 kg/m^2^ among both open and robotic transplant groups allowed for a more balanced and inclusive comparison across surgical approaches, minimizing baseline differences that might confound observational comparisons and isolating the effect of the surgical technique on postoperative pain and opioid use more effectively.

Our findings indicate that patients who remained intubated after kidney transplantation had 52.8% lower total inpatient opioid consumption within 30 days postoperatively, with robotic patients more likely to remain intubated. Current evidence suggests that prolonged intubation is associated with increased opioid exposure, which, in turn, contributes to persistent postoperative opioid use. Prior research has shown that prolonged mechanical ventilation among medical ICU patients receiving higher daily doses of opioids is associated with opioid prescriptions one year after discharge [26]. This is further supported by a study of postoperative ICU patients, which found that extubation on postoperative day (POD) 2, compared to POD 1, resulted in increased agitation or over-sedation after extubation. Furthermore, POD 2 extubation was identified as an independent risk factor for in-hospital mortality [27]. In the context of kidney transplant recipients, a prescription opioid use in the first year post-transplant is associated with a two-fold increased risk of death and a 68% increased risk of all-cause graft failure within the following year [12]. This heightened risk has been attributed to pain related to comorbidities and risky patient behaviors, which may be presented as non-compliance with immunosuppressive medications and missed clinic appointments.

Given our findings of increased opioid use associated with prolonged intubation and worsened transplant outcomes related to extended opioid use, it is essential to implement multimodal analgesia to minimize opioid exposure in both acute and chronic pain management settings. A recent quality improvement initiative by Carcella et al. demonstrates that ultrasound-guided transversus abdominis plane (TAP) or quadratus lumborum (QL) blocks in kidney transplantation significantly reduce in-hospital morphine milligram equivalents (MME) and outpatient opioid prescriptions [12]. We have also recently demonstrated that erector spinae plane (ESP) blocks in our institution reduce opioid requirements in bariatric surgery patients [28]. This approach is further supported by recommendations from the 2026 ASA practice guidelines for abdominal procedures [29], while the PROSPECT guidelines for pain control recommend a combination of fascial plane blocks and/or catheter-based regional anesthesia for various abdominal surgeries [30]. Alternative methods, such as continuous wound infusion of local anesthetics, have been reported, but conflicting results concerning opioid consumption and severe complications, including local anesthetic systemic toxicity, raise concerns [31–33]. Additional considerations may involve increasing intraoperative fentanyl dosing as a strategy to decrease long-term opioid use and prevent chronic pain diagnoses [34].

Furthermore, factors specific to robotic surgery, such as pneumoperitoneum, may contribute to postoperative pain and potentially offset the analgesic benefits of smaller incisions. A Cochrane review reports that shoulder-tip pain following laparoscopy occurs in 35% to 80% of patients and is attributed to phrenic nerve irritation caused by pneumoperitoneum, and has been associated with increased analgesic requirements, delayed recovery, prolonged hospital stay, and, in rare cases, hospital readmission [35]. Beyond its effects on pain, pneumoperitoneum may also influence respiratory physiology. In our cohort, patients undergoing robotic transplant were more likely to remain intubated at the conclusion of the procedure despite propensity matching, suggesting this finding is unlikely to be explained by baseline comorbidity differences and may instead reflect the physiologic effects of pneumoperitoneum and positioning on respiratory mechanics. Prior literature has similarly demonstrated increased ventilatory demands during robotic surgery, supporting this interpretation [36]. Strategies such as low-pressure insufflation (shown in multiple RCTs to reduce postoperative pain) [37], thorough gas evacuation [38], and intraperitoneal local anesthetic administration [39] may help mitigate this effect and warrant further investigation. In this context, pneumoperitoneum-related pain coupled with delayed extubation may partially explain the absence of reduced postoperative pain scores or opioid requirements in the robotic transplant group.

Beyond postoperative pain requirements, we investigated a number of other outcomes with almost no differences between groups. While data on other outcomes remains limited, a recent systematic review highlighted economic implications as a barrier to acquiring robotic platforms and, consequently adopting of minimally invasive surgery [40]. In addition, studies on robotic procedures have shown shorter lengths of stay, lower readmission rates, and fewer wound complications, suggesting that the perceived higher costs of robotic procedures may be offset by reduced postoperative care expenses [10, 41]. Although our findings indicate comparable outcomes in these areas, estimated blood loss and necessity for pRBC transfusions were significantly lower in the robotic transplant population. This may serve as an indirect marker of increased surgical complexity and tissue trauma, which may contribute to higher opioid consumption in the immediate postoperative period [42].

Ultimately, these findings suggest that the analgesic benefits of minimally invasive surgery may depend on optimization of perioperative care pathways. In this context, ERAS protocols may play an important role in improving recovery efficiency and potentially reducing opioid consumption and length of stay following robotic transplant [43]. Optimizing perioperative management—including early pain control and multimodal analgesia strategies such as regional anesthesia [44], including fascial plane blocks [45] and intrathecal morphine [46]—may improve postoperative outcomes. Such approaches may be particularly relevant in robotic-assisted procedures, where pneumoperitoneum-referred pain may contribute to postoperative discomfort that is not addressed by incision size alone. Additionally, incorporating postoperative physiologic measures, such as duration of mechanical ventilation and sedative-analgesic use, alongside intraoperative measures, may provide further insight into the perioperative burden associated with robotic transplant. Lastly, while larger randomized controlled trials are warranted to better define outcomes in robotic transplant surgery, widespread adoption will depend on improved access to robotic platforms and structured training programs and should be prioritized if cost equivalence can be demonstrated.

Our study possesses several strengths as well as important limitations that warrant consideration. Notably, this represents one of the larger single-center analyses comparing robotic and open transplant in a propensity-matched cohort, capturing outcomes in recipients with a mean BMI of 40 mg/kg^2^, a population frequently unevaluated for transplantation. The use of multivariable regression further strengthens our findings by identifying independent predictors of postoperative opioid use beyond surgical approach alone. There are also several limitations. First, the retrospective, single-center design limits the generalizability of our findings and introduces the potential for unmeasured confounding. Although propensity matching was performed to balance observed covariates, CAD did not achieve adequate balance (SMD = 0.22, **Supplementary Table 2**). Sensitivity analysis, with and without CAD, was performed to demonstrate that the residual imbalance does not account for our findings. Additionally, confounding from unmeasured factors such as preoperative opioid use, pain sensitivity, living and deceased donor transplants, and socioeconomic determinants may still influence postoperative opioid requirements. Remifentanil and epidural hydromorphone were excluded from the total inpatient opioid consumption within 30 days calculation, as their context-sensitivity infusion kinetics and neuraxial administration prevent meaningful conversion to systemic morphine equivalents. 30-day inpatient opioid consumption was not censored at hospital discharge, capturing any administered during readmission during that window. Lastly, variability in anesthetic and analgesic practices across providers in the intraoperative and intensive care units was not fully captured and may have contributed to differences in postoperative pain management.

The robotic transplant cohort was smaller than the open transplant cohort prior to matching, which may limit statistical power to detect differences in certain outcomes. Furthermore, postoperative pain scores were collected across multiple clinical settings and may be subject to measurement variability. Finally, while our matched design improves internal validity, it may also limit generalizability by reflecting a more selected, higher-risk patient population that may not represent all patients undergoing kidney transplantation.

## Conclusion

In summary, this propensity-matched cohort study found that robotic transplants demonstrated similar anesthetic outcomes to open transplants, including comparable postoperative opioid consumption and pain scores. Although robotic transplant demonstrated the advantage of lower intraoperative blood loss and pRBC transfusion volume, surgical approach was not an independent determinant of postoperative opioid use; this was instead more strongly influenced by patient-specific and intraoperative factors resulting in delayed extubation. Taken together, these findings confirm the feasibility of robotic approaches and identify novel pathways to improve future outcomes, like ERAS pathways for robotic cases, including protocols to accelerate extubation. These strategies may incorporate regional nerve blocks and multimodal pain control to facilitate early extubation and ambulation. Robotic transplants also demonstrated significant advantages by reducing blood loss. In order to design targeted ERAS pathways, procedural factors must be considered, including pneumoperitoneum, operative positioning, and comorbidity burden that may contribute to postoperative pain and delay recovery. These results support a more nuanced interpretation of the analgesic benefits of minimally invasive transplantation and underscore the importance of optimizing perioperative management strategies such as multimodal analgesia and ERAS pathways to reduce opioid requirements and improve recovery. Further prospective, multi-institutional studies incorporating standardized analgesic protocols are needed to better define the role of postoperative pain management in robotic transplantation and its impact on longer-term outcomes.

## Supplementary Information

Below is the link to the electronic supplementary material.

Supplementary Material 1

Supplementary Material 2

## Data Availability

All data supporting the findings of this study are available within the paper and its Supplementary Information.
