# Supplementary material for: Anesthetic outcomes in robotic kidney transplantation: a retrospective cohort analysis
Source: J Robot Surg. 2026 Nov 13;20(1):1214. doi: 10.1007/s11701-026-04168-w (PMC13768382; doi:10.1007/s11701-026-04168-w)
Supplement: Supplementary file 1 [file 11701_2026_4168_MOESM1_ESM.pdf]

Supplementary Table 1. Opioid Conversion Factors Used to Calculate Total Inpatient Opioid Consumption within 30 Days

Medication and formulation	Unit	Conversion factor	MME per unit
**Oral formulations**[a]			
Acetaminophen–codeine 300/30 mg	tablet	0.15 per mg	4.5
Hydrocodone 5 mg	tablet	1.0 per mg	5
Hydrocodone 10 mg	tablet	1.0 per mg	10
Hydromorphone 4 mg	tablet	5.0 per mg	20
Methadone 5 mg	tablet	4.7 per mg	23.5
Morphine ER 15 mg	tablet	1.0 per mg	15
Oxycodone 5 mg	tablet	1.5 per mg	7.5
Tramadol 25 mg	half tablet	0.2 per mg	5
Tramadol 50 mg	tablet	0.2 per mg	10
**Parenteral formulations**[b]			
Fentanyl 1000 mcg/100 mL (10 mcg/mL)	mL	0.2 per mcg	2.0
Fentanyl 50 mcg/mL[c]	mL	0.2 per mcg	10.0
Hydromorphone 0.2 mg/mL[d]	mL	17.5 per mg	3.5
Hydromorphone 1 mg/mL	mL	17.5 per mg	17.5
Morphine 2 mg/mL	mL	3.0 per mg	6.0
Morphine 4 mg/mL	mL	3.0 per mg	12.0

All doses are expressed in oral morphine milligram equivalents (MME). Total inpatient opioid consumption within 30 days was calculated as the sum, across all administrations, of the administered quantity multiplied by the MME per unit shown above. Calculation excludes remifentanil and epidural hydromorphone, as their context-sensitivity infusion kinetics and neuraxial administration prevent meaningful conversion to systemic morphine equivalents.

[a]*Oral formulations were converted using the NIH HEAL Initiative 2024 conversion factors (Adams et al., Pain 2025).*

[b]*Because the NIH HEAL table specifies factors for oral formulations, parenteral doses were converted using the recommended oral morphine equivalent factors of Nielsen et al. (Pharmacoepidemiol Drug Saf 2016;25:733–737): intravenous morphine 3.0 per mg, intravenous hydromorphone 17.5 per mg, and intravenous fentanyl 0.2 per microgram.*

[c]*Includes fentanyl 100 mcg/2 mL, 250 mcg/5 mL, 1000 mcg/20 mL, and 2500 mcg/50 mL, all of which are 50 mcg/mL.*

[d]*Includes hydromorphone 0.4 mg/2 mL for intravenous administration, which is 0.2 mg/mL.*

ER = extended release; MME = morphine milligram equivalents.

Supplementary Table 2. Demographics and Clinical Characteristics Before and After Propensity-Score Matching

Preoperative Characteristics	Before matching					After matching					
	**Total (N=1379)**	**Open (n=1234)**	**Robotic (n=145)**	**SMD**	***p-value***	**Total (N=254)**	**Open (n=127)**	**Robotic (n=127)**	**SMD**	***p-value***	***Discord-ant pairs***
Age (years), mean (SD)	50.7 (12.5)	51.3 (12.7)	45.6 (9.9)	−0.57	<0.001	46.4 (9.8)	46.3 (10.2)	46.4 (9.5)	0.02	0.88	
Sex, Male, % (n)	60.6% (835)	60.8% (750)	58.6% (85)	−0.04	0.65	57.9% (147)	59.8% (76)	55.9% (71)	−0.08	0.54	67
Race, % (n)					<0.001					>0.99	
Black or African American	41.0% (565)	39.2% (484)	55.9% (81)	0.34		55.9% (142)	55.9% (71)	55.9% (71)	0.00		
White	49.9% (688)	51.1% (630)	40.0% (58)	−0.23		40.2% (102)	40.2% (51)	40.2% (51)	0.00		
Other	9.1% (126)	9.7% (120)	4.1% (6)	−0.28		3.9% (10)	3.9% (5)	3.9% (5)	0.00		
Weight (kg), median (IQR)	85.0 (69.6–106.3)	81.6 (68.0–101.2)	121.6 (106.3–136.2)	1.68	<0.001	117.9 (104.5–132.9)	117.3 (104.7–133.2)	119.1 (104.9–132.7)	0.00	0.86	
BMI (kg/m²), median (IQR)	30.3 (25.2–36.2)	28.9 (24.8–34.4)	40.8 (36.7–44.4)	1.87	<0.001	40.1 (36.4–43.4)	40.1 (36.2–43.6)	40.0 (36.5–43.1)	0.05	0.61	
ASA Score, % (n)					0.19					0.59	
II	0.5% (7)	0.4% (5)	1.4% (2)	0.08		0.4% (1)	0.0% (0)	0.8% (1)	0.07		
III	87.3% (1204)	87.2% (1076)	88.3% (128)	0.03		89.0% (226)	89.0% (113)	89.0% (113)	0.00		
IV	12.2% (168)	12.4% (153)	10.3% (15)	−0.07		10.6% (27)	11.0% (14)	10.2% (13)	−0.03		
Comorbidities, % (n)											
HTN	28.7% (396)	29.7% (366)	20.7% (30)	−0.22	0.03	20.1% (51)	19.7% (25)	20.5% (26)	0.02	0.88	41
HLD	33.4% (461)	33.0% (407)	37.2% (54)	0.09	0.31	37.4% (95)	37.8% (48)	37.0% (47)	−0.02	0.90	63
DM	49.5% (682)	49.1% (606)	52.4% (76)	0.07	0.48	54.7% (139)	55.1% (70)	54.3% (69)	−0.02	0.90	67
CAD	19.4% (268)	20.3% (250)	12.4% (18)	−0.23	0.03	9.8% (25)	6.3% (8)	13.4% (17)	0.22*	0.09	23
CHF	16.3% (225)	16.7% (206)	13.1% (19)	−0.11	0.34	16.1% (41)	17.3% (22)	15.0% (19)	−0.07	0.59	31

OSA	19.2% (265)	16.5% (204)	42.1% (61)	0.52	<0.001	37.8% (96)	37.8% (48)	37.8% (48)	0.00	>0.99	62
Anemia	44.5% (613)	44.2% (546)	46.2% (67)	0.04	0.66	45.7% (116)	45.7% (58)	45.7% (58)	0.00	>0.99	84

ASA = American Society of Anesthesiologists; BMI = body mass index; CAD = coronary artery disease; CHF = congestive heart failure; DM = diabetes mellitus; HLD = hyperlipidemia; HTN = hypertension; OSA = obstructive sleep apnea; SD = standard deviation; SMD = standardized mean difference.

Data are mean (SD), median (IQR), or % (n). SMD calculated for continuous and binary covariates using the standard deviation of the pre-matching robotic group as a common denominator; |SMD| < 0.10 indicates adequate balance.

Before matching: independent-samples t-test (age), Wilcoxon rank-sum test (weight, BMI), Fisher's exact test (categorical). After matching: paired t-test (age), Wilcoxon signed-rank test (weight, BMI), McNemar's test (binary covariates; exact binomial form where <25 discordant pairs), Stuart–Maxwell test of marginal homogeneity (race, ASA).

ASA physical status was entered into the propensity model as a numeric score; balance is reported here by category.

*Residual imbalance after matching (|SMD| ≥ 0.10). Sensitivity analyses adjusting for coronary artery disease are reported in the text.

Supplementary Table 3. Sensitivity Analyses of the Association Between Surgical Approach and Total Inpatient Opioid Consumption within 30 Days (MME)

Model	Patients	Complete pairs	Ratio of geometric means (95% CI)	% change in 30d MME (95% CI)	*p-value*
Baseline covariates[a]	253	126	1.11 (0.91 to 1.36)	11.1% (−9.1% to 35.7%)	0.30
Baseline covariates + coronary artery disease[b]	253	126	1.13 (0.93 to 1.39)	13.3% (−7.4% to 38.5%)	0.22
Baseline covariates + all comorbidities[c]	253	126	1.15 (0.94 to 1.40)	15.0% (−5.9% to 40.5%)	0.17
Baseline covariates, cluster-robust SE by matched pair[d]	253	126	1.11 (0.90 to 1.38)	11.1% (−10.2% to 37.5%)	0.33
Model additionally adjusted for intraoperative and postoperative variables[e]	195	76	0.99 (0.78 to 1.27)	−0.5% (−22.1% to 27.1%)	0.97

Estimates represent the association between robotic transplantation (reference: open) and total inpatient opioid consumption within 30 days. The outcome was log-transformed prior to multivariable linear regression to improve normality of residuals; coefficients are presented exponentiated, as ratios of geometric means and percent difference. A ratio above 1.00 indicates higher opioid consumption in the robotic group.

Models restricted to pre-operative covariates were fitted on 253 of 254 matched patients, comprising 126 complete matched pairs and 1 patient whose matched counterpart was missing outcome data.

[a]*Baseline covariates: surgical approach, age, sex, race, weight, body mass index, and ASA physical status. Only pre-operative covariates were included, so that estimates represent the total association of surgical approach with opioid consumption.*

[b]*Baseline covariates with coronary artery disease added, the single covariate with a residual standardized mean difference ≥ 0.10 after matching (Table 1).*

[c]*Baseline covariates with coronary artery disease, hypertension, hyperlipidemia, diabetes mellitus, congestive heart failure, obstructive sleep apnea, and anemia added.*

[d]*Baseline covariate model refitted with standard errors clustered on matched pair, to account for dependence between the members of each pair.*

[e]*Baseline covariates with procedure duration, intraoperative morphine milligram equivalents, estimated blood loss, extubation status, and highest pain score within 72 hours added (the model reported in Table 3). These covariates occur during or after surgery and may lie on the causal pathway between surgical approach and opioid consumption; this estimate is therefore conditional on those variables and does not represent the total association of surgical approach. The reduced sample reflects missing data on these covariates.*

CI = confidence interval; MME = morphine milligram equivalents; SE = standard error.
